# Tibolone Administration Is Associated with Enhanced Motor Recovery and Decreased Cell-Specific NOX2 and NOX4 Immunoreactivity in a Rat Model of Traumatic Spinal Cord Injury

**DOI:** 10.3390/brainsci16070711

**Published:** 2026-07-01

**Authors:** Tzayaka Castillo-Mendieta, Stephanie Sánchez-Torres, Hermelinda Salgado-Ceballos, Julia J. Segura-Uribe, Julio Morán, Christian Guerra-Araiza, Angélica Coyoy-Salgado

**Affiliations:** 1 Unidad de Investigación Médica en Farmacología, Hospital de Especialidades Dr. Bernardo Sepúlveda, Centro Médico Nacional Siglo XXI, Instituto Mexicano del Seguro Social, Mexico City 06720, Mexico; 2 Unidad de Investigación Médica en Enfermedades Neurológicas, Hospital de Especialidades Dr. Bernardo Sepúlveda, Centro Médico Nacional Siglo XXI, Instituto Mexicano del Seguro Social, Mexico City 06720, Mexico; 3 Departamento de Investigación Traslacional en Farmacoepidemiología, Hospital Infantil de México Federico Gómez, Secretaría de Salud, Mexico City 06720, Mexico; drjuseur.farmaepid@gmail.com; 4 División de Neurociencias, Instituto de Fisiología Celular, Universidad Nacional Autónoma de México, Mexico City 04510, Mexico

**Keywords:** tibolone, spinal cord injury, NOX2, NOX4, oxidative stress, motor recovery

## Abstract

**Highlights:**

**What are the main findings?**

- Tibolone (TIB) modulates neuronal NOX2 localization in a dose- and time-dependent manner after spinal cord injury (SCI).
- NOX4 localization remains largely unaffected, decreasing only in astrocytes at higher TIB doses.
- TIB treatment at 2.5 mg/kg improved motor recovery in SCI rats compared to untreated controls.

**What are the implications of the main findings?**

- TIB treatment was associated with changes in NOX2 and NOX4 immunoreactivity patterns in a cell-specific and time-dependent manner following SCI.
- Dose-dependent cellular responses to TIB underline the need for precise dosing strategies to optimize therapeutic windows and cellular outcomes in experimental SCI models.

**Abstract:**

Background/Objectives: This study aimed to investigate the effect of tibolone (TIB) on the expression of the NOX2 and NOX4 isoforms and their co-localization in neurons and astrocytes using an animal model of spinal cord injury (SCI). Methods: Male Sprague Dawley rats were subjected to contusive SCI at T9 and treated with TIB (1 or 2.5 mg/kg/day). The expression of NOX2 and NOX4, and their co-localization in neurons and astrocytes, were evaluated at 3, 7, and 15 days post-SCI. Oxidative stress markers and motor recovery were also assessed. Results: SCI induced a time-dependent increase in NOX2 protein expression at 15 days compared to 3 days (0.192 ± 0.012 vs. 0.013 ± 0.0007) and in NOX4 protein expression (0.027 ± 0.002 vs. 0.958 ± 0.088), displaying differential cell-specific localization patterns in neurons and astrocytes across time points. TIB treatment significantly attenuated SCI-induced oxidative stress (15,391.8 ± 1047.6 vs. 21,264.4 ± 2669.8) and decreased NOX2 and NOX4 co-localization in both neurons and astrocytes in a dose- and time-dependent manner. Furthermore, TIB-treated animals exhibited a modest but significant restoration of motor function at 15 days post-SCI. Conclusions: Our findings indicate that TIB administration reduces oxidative stress, modifies NOX2 and NOX4 immunoreactivity patterns in neurons and astrocytes, and promotes partial functional recovery following SCI.

## 1. Introduction

Spinal cord injury (SCI) can result in permanent autonomic, sensory, and motor dysfunction, not only from immediate mechanical damage during trauma but also from a series of secondary injury mechanisms [1]. These mechanisms cause cellular dysfunction and death through processes such as ischemia, edema, ionic imbalance, glutamate-mediated excitotoxicity, mitochondrial dysfunction, metabolic inhibition, and oxidative damage [2].

Additionally, SCI triggers an acute and sustained inflammatory response characterized by immune cell infiltration and a significant increase in reactive oxygen species (ROS) production [3]. The nicotinamide adenine dinucleotide phosphate (NADPH) oxidase (NOX) is a family of enzymes primarily responsible for ROS production in the injured spinal cord [4,5,6].

The enzymes in the NOX family are transmembrane carriers that convert oxygen into superoxide anion by transferring an electron from cytosolic NADPH [7]. NOX-1, NOX-2, NOX-3, NOX-4, NOX-5, dual oxidase-1 (DUOX-1), and DUOX-2 are the seven known members that combine with different subunits to form active enzyme complexes. Both NOX and DUOX isoforms share structural similarities, including six conserved transmembrane helices and binding sites for heme, nicotinamide adenine dinucleotide phosphate, and flavin adenine dinucleotide [8].

Experimental studies have demonstrated that NOX isoforms are present in the brain and spinal cord across different cell types [4,9,10]. In the spinal cord, NOX2 is mainly found in microglia and neurons, NOX3 is primarily expressed in neurons, and NOX4 is present in all three major cell types (neurons, astrocytes, and microglia) [4,9,10]. Khayrullina et al. (2023) reported that both NOX2 and NOX4 contribute to ROS production after SCI; however, only NOX2 genetic knockout inhibited ROS production, leading to sustained functional recovery and decreased chronic inflammation [6].

Given the multifactorial nature of SCI progression, more effective therapeutic strategies are urgently needed [11]. Studies have shown that estrogen and its derivatives protect against functional impairments after SCI. Estrogen has been shown to improve scores on the Basso, Beattie, Bresnahan (BBB) locomotor rating scale [12,13]. Additionally, estradiol (E2) significantly reduces neuronal NADPH oxidase activation and superoxide (O_2_^−^) production in the CA1 region following global cerebral ischemia (GCI) and reperfusion, suggesting that some of E2′s neuroprotective effects depend on early modulation of oxidative stress pathways and NOX signaling [14].

However, the increased risk of breast and endometrial cancer associated with estrogen therapy has led to the development of synthetic steroids like tibolone (TIB) [15]. TIB mainly acts depending on the tissue and can produce estrogenic, androgenic, or progestogenic effects [16]. During metabolism, TIB is transformed into three metabolites: 3α-hydroxytibolone and 3β-hydroxytibolone, which have estrogenic activity by activating ERα and ERβ, and the Δ4-isomer metabolite formed by reducing the double bond in TIB’s A-ring [17,18].

Several studies have shown that TIB provides protective effects in both astrocytes and neurons by reducing oxidative stress and enhancing mitochondrial function after metabolic damage and lipotoxicity [19]. Recently, we demonstrated that TIB administration in a rat model of SCI exerts neuroprotective effects in SCI through multiple mechanisms [20,21,22]. TIB administration reduced structural damage to neural tissue, preserved the surrounding tissue, and modulated autophagy markers in a time-dependent manner while consistently inhibiting apoptosis [20]. Moreover, TIB regulated pro- and anti-inflammatory cytokine levels across the early, subacute, and chronic phases post-SCI, and prolonged oral administration reduced gliosis while promoting tissue preservation and functional recovery [21,22].

Additionally, TIB decreased oxidative stress markers (malondialdehyde and protein carbonylation) early after SCI [21]. However, ROS regulation by TIB remains insufficiently characterized. To date, no studies have examined whether TIB modulates NADPH oxidase isoforms, specifically NOX2 and NOX4, which are critical contributors to oxidative stress following SCI. Addressing this knowledge gap is essential, as targeting NOX-dependent pathways may open new avenues for therapeutic intervention. Accordingly, the present study investigated the effects of TIB on the protein expression of NOX2 and NOX4, and on their co-localization, in neurons and astrocytes using a contusion-based rat model of SCI.

## 2. Materials and Methods

### 2.1. Animals and Surgical Procedures

Male Sprague Dawley rats (250–300 g) were maintained under controlled environmental conditions (12-h light:12-h dark cycle) with unrestricted access to food and water. Before surgical procedures, animals were randomly allocated to experimental groups using a computer-generated sequence (simple randomization). Group assignment was performed by an investigator independent of the surgeries and outcome assessments to minimize selection bias. Rats were distributed into three groups: SCI (vehicle control), SCI + TIB 1 (1 mg/kg), and SCI + TIB 2.5 (2.5 mg/kg).

Independent cohorts of animals were used for each experimental technique and time point. For histological and Western blot analyses, 4–5 rats per group were included at each time point (3, 7, and 15 days post-injury). For functional recovery assessments, 8 rats per group were analyzed. Importantly, distinct sets of animals were employed for each experimental endpoint to avoid cross-contamination of data and ensure methodological integrity.

For surgical procedures, animals were anesthetized intramuscularly with a mixture of xylazine (75 mg/kg; Xylazine, PiSA, Guadalajara, Mexico) and Zoletil (25 mg/kg; Zoletil 100, Virbac, Carros, France). A laminectomy was performed at the level of thoracic vertebra 9 (T9), followed by induction of SCI using the New York University (NYU) impactor (New York University, NY, USA) [23]. The SCI was produced by dropping a 10-g rod from a height of 25 mm [24]. Hematoma formation at the lesion site was confirmed by microscopic observation. Subsequently, the muscle and skin layers were sutured sequentially.

An antibiotic (benzathine penicillin, 1,200,000 IU; PiSA, Guadalajara, Mexico) was administered as a single intramuscular dose, and an analgesic (paracetamol, 5 mL/L of drinking water) was provided for five consecutive days. Following surgery, animals were housed individually under the previously described controlled conditions. Neurogenic bladder and bowel functions were manually expressed daily until sphincter control was regained. The surgical wound and overall health status of each animal were monitored daily to ensure postoperative care and welfare.

Healthy male Sprague Dawley rats weighing 250–300 g at the time of surgery were included in the study. Only animals that successfully underwent induction of SCI at the T9–T10 level using the NYU impactor were analyzed. Exclusion criteria comprised death during or immediately after surgery, absence of a clear contusion injury following the impactor procedure, or postoperative complications unrelated to the experimental model that impaired survival or recovery. Notably, no animals were excluded due to treatment effects.

### 2.2. Treatments

Animals received the assigned treatment 30 min after surgery and then every 24 h for 3, 7, and 15 days. Animals in the SCI groups were orally administered TIB or vehicle (water). Tibolone (TIB; Livial^®^, 2.5 mg, Organon, Oss, The Netherlands) was prepared in water and administered via an esophageal cannula to the treatment groups. For the TIB 1 mg/kg dose, one tablet was dissolved in 2.5 mL of water, and the volume was calculated based on body weight (e.g., 250 μL for a 250 g rat). For the TIB 2.5 mg/kg dose, one tablet was dissolved in 1 mL of water, and the volume was calculated based on body weight (e.g., 250 μL for a 250 g rat). TIB doses (1 mg/kg/day or 2.5 mg/kg/day) were selected based on previous studies demonstrating TIB’s neuroprotective effects, including improved memory, antioxidant properties, and functional motor recovery [16,20,25,26,27,28]. After the treatment period, animals were euthanized in accordance with the Official Mexican Standards (NOM) for humane euthanasia of animals [29,30].

### 2.3. Tissue Collection

At the end of each treatment period, all animals were sacrificed. For immunofluorescence analysis, animals were anesthetized with pentobarbital and perfused intracardially with saline, followed by 4% paraformaldehyde at a flow rate of 30 mL/min via a peristaltic pump. After perfusion, a 2-cm segment of the spinal cord was extracted, centered on the lesion epicenter, with 1 cm of tissue preserved rostrally and caudally.

Tissues were post-fixed in 4% paraformaldehyde for 8 days, then dehydrated through a graded ethanol series (70%, 96%, and 100%), followed by xylene and paraffin processing, with each step lasting 15 min. Samples were embedded in paraffin blocks in a ventral–dorsal orientation. Transverse spinal cord sections, 5 µm thick, were obtained with a microtome (RM2125 RTS, Leica Biosystems, Deer Park, IL, USA) and mounted on poly-L-lysine-coated glass slides. Representative sections were selected from each animal using the lesion epicenter and the ependymal canal as anatomical reference points.

For Western blot analysis, animals were euthanized at 3, 7, and 15 days after SCI, and spinal cord tissue was collected on ice. A 1 cm segment centered on the lesion epicenter was isolated, immediately frozen in liquid nitrogen, and stored at −80 °C until protein extraction for Western blot analysis.

### 2.4. Immunofluorescence Analysis

Selected slides were incubated in an oven at 60 °C to remove residual paraffin. Samples were then deparaffinized through a graded ethanol series and subjected to antigen retrieval by placing them in 10 mM citrate buffer (pH 6.0) in a plastic Coplin jar, followed by heating in a pressure cooker for 20 min. After cooling, tissue sections were permeabilized in PBS-T (0.01 M PBS with 0.1% Triton X-100) for 30 min. Non-specific binding sites were blocked by incubating the samples with 5% horse serum in PBS for 30 min in a humidified chamber.

Tissues were incubated overnight at 4 °C with the following primary antibodies diluted in blocking solution: goat anti-4-hydroxynonenal (4-HNE) (1:500, Merck KgaA, Darmstadt, Germany), mouse anti-gp91-phox (1:50, No. Cat. 130548, Santa Cruz Biotechnology Inc., Dallas, TX, USA), rabbit anti-NADPH oxidase 4 (1:750, No. Cat. 155071, Abcam, Cambridge, UK), rabbit anti-GFAP (1:500, No. Cat. AHP669, Bio-Rad Laboratories Inc., Hercules, CA, USA), mouse anti-NeuN (1:500, No. Cat. 279296, Abcam, Cambridge, UK), and rabbit anti-NeuN (1:500, No. Cat. D4G40, Cell Signaling, Danvers, MA, USA). After primary antibody incubation, slides were washed with PBS and incubated for 2 h at room temperature in the dark with the following secondary antibodies: Alexa Fluor^®^ 488 donkey anti-rabbit IgG (1:300, Molecular Probes, Eugene, OR, USA), Alexa Fluor^®^ 594 donkey anti-mouse IgG (1:300, Molecular Probes, Eugene, OR, USA), and Alexa Fluor^®^ 594 donkey anti-goat IgG (1:500, Molecular Probes, Eugene, OR, USA).

Cell nuclei were counterstained with DAPI (1 μM) for 2 min. To reduce background fluorescence, sections were incubated with 0.1% Sudan Black B (Sigma-Aldrich, St. Louis, MI, USA) in 70% ethanol for 15 min, then rinsed with PBS. Coverslips were mounted with Vectashield^®^ mounting medium (Vector Laboratories, CA, USA).

Fluorescence images were acquired using a Fluoview FV1000 confocal microscope (Olympus, Tokio, Japan) and processed with FV10-ASW 1.6 software (Olympus, Tokio, Japan). Quantitative analyses were performed on spinal cord sections, with the lesion epicenter serving as the anatomical reference. Cell colocalization was quantified within a 1-cm segment encompassing the rostral region, lesion epicenter, and caudal region. Cells exhibiting colocalization of HNE/DAPI, NeuN/NOX2, NeuN/NOX4, GFAP/NOX2, and GFAP/NOX4 were manually counted. All image acquisition and quantitative analyses were conducted by two investigators blinded to group allocation to ensure objectivity. Manual quantification of colocalizing cells was performed using FIJI (ImageJ, version 1.38×).

### 2.5. Western Blot Analysis

Spinal cord tissue samples were homogenized in an ice-cold lysis buffer (pH 7.4) containing 25 mM Tris, 50 mM NaCl, 2% Igepal, 0.2% SDS, and a protease inhibitor cocktail. Homogenates were then centrifuged at 15,000× *g* for 30 min at 4 °C, and the resulting supernatants were collected and stored at −20 °C until further analysis. Protein concentration was measured with the Bradford assay.

For quantifying protein levels, samples from independent animals were analyzed. Equal amounts of total protein were separated by SDS-PAGE and transferred to polyvinylidene fluoride (PVDF) membranes (Millipore, Burlington, MA, USA). Membranes were blocked for 1 h at room temperature with 5% non-fat dry milk in Tris-buffered saline (TBS: 100 mM Tris-HCl, 150 mM NaCl, pH 7.5). After blocking, membranes were washed three times with TBS containing 0.1% Tween-20 (TTBS) for 5 min each.

Membranes were incubated overnight at 4 °C with the following primary antibodies diluted in TTBS: anti-mouse gp91-phox (1:750, No. Cat. 130548, Santa Cruz Biotechnology, Dallas, TX, USA), rabbit monoclonal anti-NADPH oxidase 4 antibody (1:750, No. Cat. 155071, Abcam, Cambridge, UK), and GAPDH antibody (1:1000; No. Cat. Sc-32233; Santa Cruz Biotechnology, Dallas, TX, USA), which served as the internal loading control. After primary antibody incubation, membranes were washed three times with TTBS for 5 min each and then incubated with the appropriate horseradish peroxidase (HRP)-conjugated secondary antibodies: anti-mouse IgG (1:10,000; cat. 115-035-003, Jackson ImmunoResearch Laboratories, Inc., West Grove, PA, USA) or anti-rabbit IgG (1:10,000; cat. 211-032-171, Jackson ImmunoResearch Laboratories, Inc., West Grove, PA, USA) for 2 h at room temperature.

After secondary incubation, membranes were washed again (3 × 5 min) with TTBS and developed with Immobilon^®^ Crescendo Western HRP Substrate (Merck Millipore, Burlington, MA, USA) for 1 min. Chemiluminescent signals were captured with the Fusion FX imaging system (Vilber Lourmat, Eberhardzell, Germany) and analyzed with EVOLUTION-CAPT software (Vilber Lourmat, Eberhardzell, Germany). Densitometric analysis of the bands was performed with Fiji (NIH ImageJ, version 1.38).

### 2.6. Assessment of Functional Recovery

Recovery of motor function in the open field was evaluated using the Basso, Beattie, and Bresnahan (BBB) locomotor scale in a double-blind design. This scale assesses hindlimb joint movement, paw placement on the plantar surface, weight-bearing capacity, and forelimb–hindlimb coordination during locomotion. The BBB is a 21-point scale, where 0 denotes complete absence of hindlimb movement, and 21 indicates normal locomotor activity. Motor recovery was independently scored by three blinded observers to ensure objectivity and minimize bias. The first assessment was conducted 24 h post-injury to confirm complete hindlimb paralysis, followed by weekly evaluations for two weeks to monitor functional recovery [31,32].

### 2.7. Statistical Analysis

Data were analyzed using GraphPad Prism 8.0. Normality was assessed with the Shapiro–Wilk test, and homogeneity of variance was evaluated using Levene’s test. Biochemical and immunofluorescence data were analyzed by two-way analysis of variance (ANOVA) followed by Bonferroni post hoc comparisons. BBB locomotor scores were analyzed using repeated-measures one-way ANOVA, followed by Tukey’s post hoc test. Results are presented as means ± standard error (SE). In addition to *p*-values, effect sizes were estimated using Hedges’ g, and 95% confidence intervals were calculated for key comparisons to provide a more precise estimate of the magnitude and reliability of observed effects.

## 3. Results

### 3.1. Effect of TIB on Oxidative Stress-Associated Lipid Peroxidation over Time After SCI

**Figure 1:**
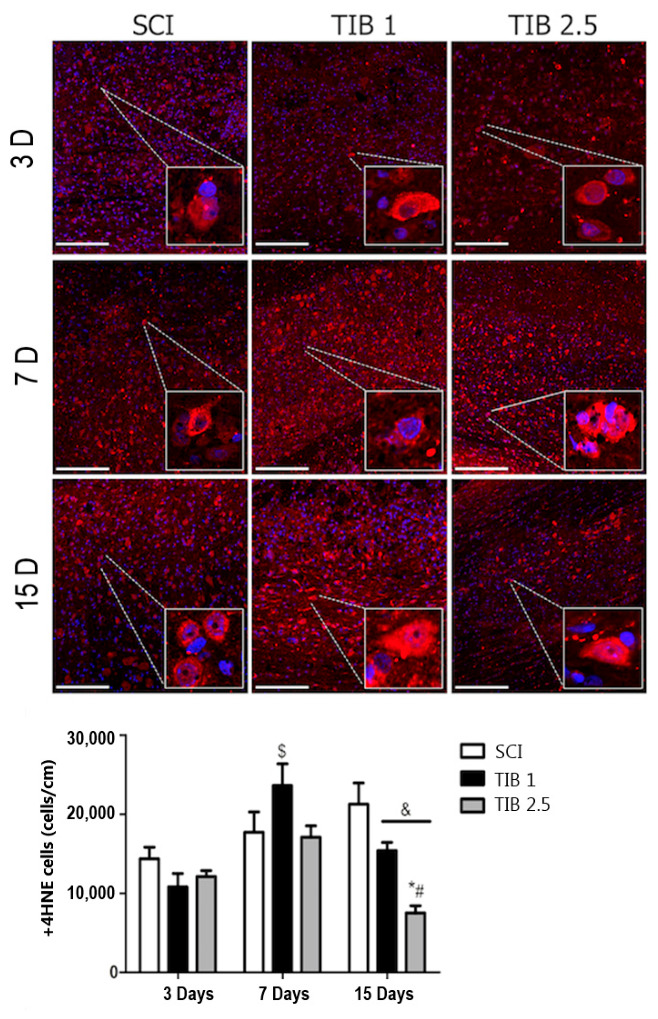
Effect of Tibolone (TIB) on oxidative stress-associated lipid peroxidation at different times after spinal cord injury (SCI). Representative images show immunohistochemical labeling of 4-hydroxynonenal (HNE, red) and nuclei (DAPI, blue) in the caudal region of the spinal cord at 3, 7, and 15 days post-SCI. 4-HNE was used as an indirect marker of lipid peroxidation and oxidative stress. Independent cohorts of animals were used for each time point (*n* = 4 animals per group per time point; SCI, TIB 1, and TIB 2.5). Images were acquired at 20× magnification (scale bar = 200 μm). The graph shows the number of 4-HNE-positive cells colocalized with DAPI, quantified across the cephalic, epicentral, and caudal regions within 1 cm of the spinal cord. Data are presented as means ± SE. Statistical analysis used two-way ANOVA with Bonferroni’s post hoc test (* *p* < 0.05 vs. SCI; # *p* < 0.05 vs. TIB 1; $ *p* < 0.05 vs. 3 days; & *p* < 0.05 vs. 7 days).

We evaluated oxidative stress-associated lipid peroxidation at 3, 7, and 15 days after SCI, along with the effects of TIB 1 and TIB 2.5, using 4-HNE as an indirect marker of oxidative damage. Our findings indicated that 4-HNE-positive cells were detected at all analyzed time points, indicating sustained lipid peroxidation following injury. While TIB treatment did not modify the number of 4-HNE-positive cells at 3 and 7 days post-injury, it significantly reduced oxidative stress at 15 days post-injury, but only at the highest dose tested (21,264.4 ± 2669.8 vs. 7505.8 ± 906.0; *p* < 0.05; 95% CI for the mean difference [SCI − TIB 2.5]: 6424 to 21,090; Hedges’ g = 2.18) (Figure 1).

### 3.2. Effect of TIB on NOX2 and NOX4 Protein Levels at Various Time Points After SCI

**Figure 2:**
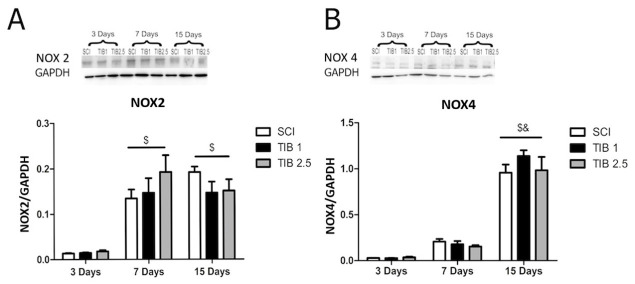
Effect of Tibolone (TIB) on NADPH oxidase 2 (NOX2) and NADPH oxidase 4 (NOX4) expression after spinal cord injury (SCI). Protein levels of (**A**) NOX2 and (**B**) NOX4 in rat spinal cords were measured at 3, 7, and 15 days after surgery in the SCI, TIB 1 (tibolone 1 mg/kg), and TIB 2.5 (tibolone 2.5 mg/kg) groups and quantified by Western blot analysis. Independent cohorts were used at each time point (*n* = 4–5 animals per group). Representative blots show NOX2 (~60 kDa), NOX4 (~67 kDa), and GAPDH (~36 kDa). Densitometric analysis of NOX2/GAPDH and NOX4/GAPDH ratios is presented as mean ± SE. Statistical analysis was performed using a two-way ANOVA followed by a Bonferroni post hoc test ($ *p* < 0.001 vs. 3 days; & *p* < 0.05 vs. 7 days).

Since NOX enzymes, particularly the NOX2 and NOX4 isoforms, are major sources of oxidative stress following SCI, we assessed the effect of TIB on NOX2 and NOX4 protein levels at different time points after SCI. NOX2 protein levels showed a time-dependent increase, with higher levels observed at 7 days (0.134 ± 0.019) and 15 days (0.192 ± 0.012) post-SCI compared to 3 days (0.013 ± 0.0007; *p* < 0.001; 95% CI for the mean difference [SCI 7D − SCI 3D]: 0.0822 to 0.2748; Hedges’ g = 8.7). Treatment with TIB did not result in statistically significant changes in NOX2 protein levels at any of the evaluated time points (Figure 2A). Similar results were observed for NOX4 protein levels, which increased at 15 days post-SCI compared to 3 days (0.958 ± 0.088 vs. 0.027 ± 0.002; *p* < 0.001; 95% CI for the mean difference [SCI 15D − SCI 3D]: 0.6320 to 1.230; Hedges’ g = 6.7). However, no statistically significant differences in NOX4 levels were observed between the TIB-treated and untreated groups at any of the evaluated time points (Figure 2B).

### 3.3. Effect of TIB on NOX2 and NOX4 Co-Localization in Neurons at 3, 7, and 15 Days After SCI

**Figure 3:**
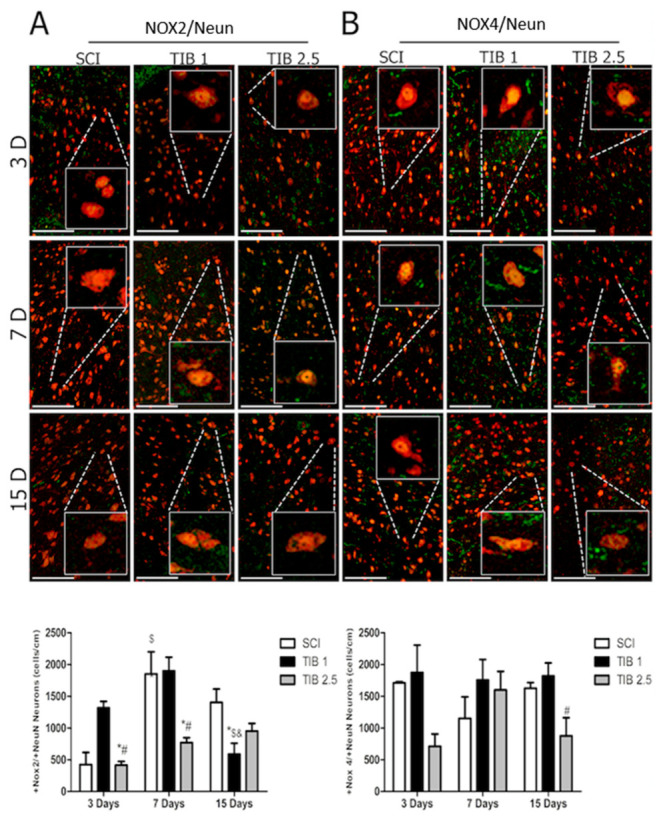
Effect of Tibolone (TIB) on NOX2 and NOX4 co-localization in neurons at 3, 7, and 15 days after SCI. (**A**) Representative images showing immunohistochemical labeling of NOX2 (green) and neurons (NeuN, red) in the caudal areas of the spinal cord at 3, 7, and 15 days post-injury in the SCI, TIB 1, and TIB 2.5 groups. (**B**) Representative images showing immunohistochemical labeling of NOX4 (green) and neurons (NeuN, red) in the caudal areas of the spinal cord at 3, 7, and 15 days post-injury in the SCI, TIB 1, and TIB 2.5 groups. Independent cohorts of animals were used for each time point (*n* = 4 animals per group per time point; SCI, TIB 1, and TIB 2.5). Images were acquired at 20× magnification (scale bar = 200 μm). Quantitative analysis shows the number of NOX2/NeuN and NOX4/NeuN colocalized cells in the cephalic, epicentral, and caudal regions of 1 cm of spinal cord. Data are presented as means ± SE Statistical analysis was performed using two-way ANOVA followed by Bonferroni post hoc test (* *p* < 0.05 vs. SCI; # *p* < 0.05 vs. TIB 1; $ *p* < 0.05 vs. 3 days; & *p* < 0.05 vs. 7 days).

To assess the localization of NOX2 and NOX4 in specific cell types, we first analyzed their localization in neurons and evaluated changes induced by SCI and TIB at various time points. Immunofluorescence analysis revealed that SCI induced a significant increase in the number of NOX2/NeuN co-localized cells only at 7 days post-SCI, compared with 3 days post-SCI. Interestingly, no changes in the number of NOX4-positive cells were observed under SCI conditions at any of the time points evaluated. At 7 days post-SCI, the TIB 2.5 group showed a significant decrease in the number of NOX2-positive neurons compared with the SCI group (770.3 ± 76.3 vs. 1850.5 ± 347.5; * *p* < 0.05; 95%; the mean difference (SCI–TIB 2.5) was 1080.2 cells (95% CI, 93.4 to 2067; Hedges’ g = 2.55), indicating a reduction in neuronal NOX2 co-localization at the highest dose. At 15 days post-SCI, the number of NOX2-positive neurons decreased only in the TIB 1 group, and this difference was statistically significant compared with the untreated SCI group (588 ± 171.7 vs. 1402.6 ± 210.4; * *p* < 0.05; 95% CI for the mean difference [SCI − TIB 1]: 101.3 to 1527; Hedges’ g = 1.36) (Figure 3A).

At 15 days post-SCI, differences were observed in the number of NOX4/NeuN co-localized cells between TIB 1 and TIB 2.5 (1820 ± 202.5 vs. 876 ± 283.7; * *p* < 0.05; 95% CI for the mean difference [TIB 1 − TIB 2.5]: 41.31 to 1847; Hedges’ g = 0.99) (Figure 3B).

### 3.4. Effect of TIB on NOX2 and NOX4 Co- Localization in Astrocytes at 3, 7, and 15 Days After SCI

**Figure 4:**
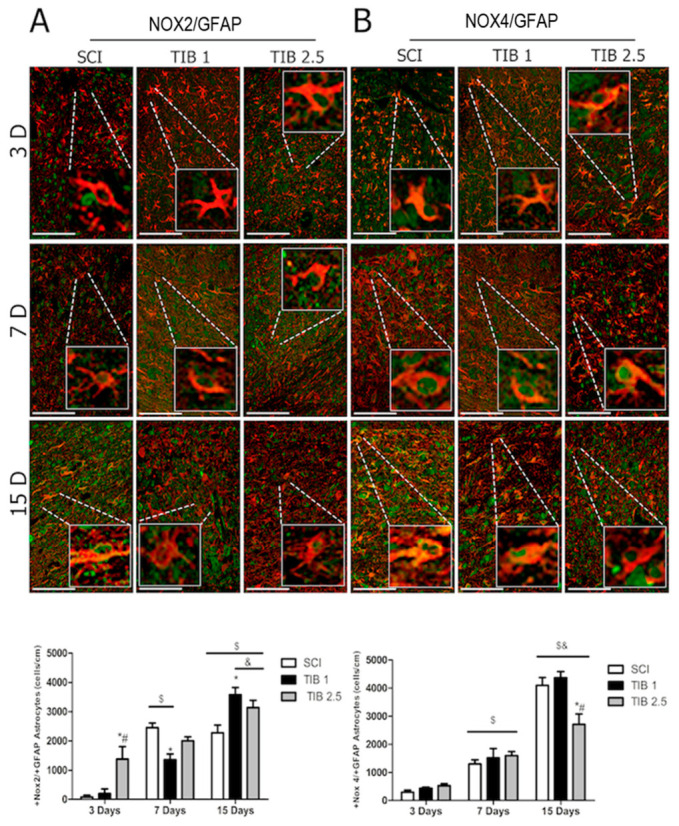
Effect of Tibolone (TIB) on NOX2 and NOX4 co-localization in astrocytes at 3, 7, and 15 days after SCI. (**A**) Representative images showing immunohistochemical labeling of NOX2 (green) and astrocytes (GFAP, red) in the caudal area of the spinal cord at 3, 7, and 15 days post-injury in the SCI, TIB 1, and TIB 2.5 groups. (**B**) Representative images showing immunohistochemical labeling of NOX4 (green) and astrocytes (GFAP, red) in the caudal area of the spinal cord at 3, 7, and 15 days post-injury in the SCI, TIB 1, and TIB 2.5 groups. Independent cohorts of animals were used for each time point (*n* = 4 animals per group per time point; SCI, TIB 1, and TIB 2.5). Images were acquired at 20× magnification (scale bar = 200 μm). Quantitative analysis shows the number of NOX2/ GFAP and NOX4/ GFAP co-localized cells in the cephalic, epicentral, and caudal regions of a 1 cm segment of spinal cord. Data are presented as means ± SE. Statistical analysis was performed using two-way ANOVA followed by Bonferroni post hoc test (* *p* < 0.05 vs. SCI; # *p* < 0.05 vs. TIB 1; $ *p* < 0.001 vs. 3 days; & *p* < 0.001 vs. 7 days).

We also examined co-localization of NOX2 and NOX4 in astrocytes and evaluated changes induced by SCI and TIB at multiple time points. Immunofluorescence analysis showed that SCI induced a significant increase in the number of NOX2/GFAP co-localized cells at 7 and 15 days after SCI (Figure 4).

Although the co-localization of NOX2 and NOX4 markedly increased in astrocytes at all time points after SCI, only a reduction in NOX2-positive astrocytes was observed in the TIB 1 group at 7 days post-injury compared with the SCI group (1357.0 ± 194.4 vs. 2450.0 ± 159.1; * *p* < 0.05; 95% CI for the mean difference [SCI − TIB 1]: 370.8 to 1815; Hedges’ g = 3.12), and in NOX4-positive astrocytes in the TIB 2.5 group at 15 days post-injury compared with the SCI group (2705.6 ± 370.4 vs. 4092.3 ± 281.9; * *p* < 0.05; 95% CI for the mean difference [SCI − TIB 2.5]: 136 to 2637; Hedges’ g = 1.84) (Figure 4). At 3 days post-SCI, the number of NOX2-positive astrocytes was significantly higher in the TIB 2.5 group compared with the untreated SCI group (1375.0 ± 432.0 vs. 81.0 ± 61.6; * *p* < 0.05; 95% CI for the mean difference [SCI − TIB 2.5]: −2283 to −304.9; Hedges’ g = 2.63) (Figure 4A). At 15 days post-SCI, the number of NOX2-positive astrocytes was significantly higher in the TIB 1 group compared to the untreated SCI group (3573.0 ± 251.9 vs. 2276.6 ± 263.8; * *p* < 0.05; 95% CI for the mean difference [SCI − TIB 1]: −2429 to −163.5; Hedges’ g = 1.88) (Figure 4A).

### 3.5. TIB Enhances Motor Function Recovery 15 Days After SCI

**Figure 5:**
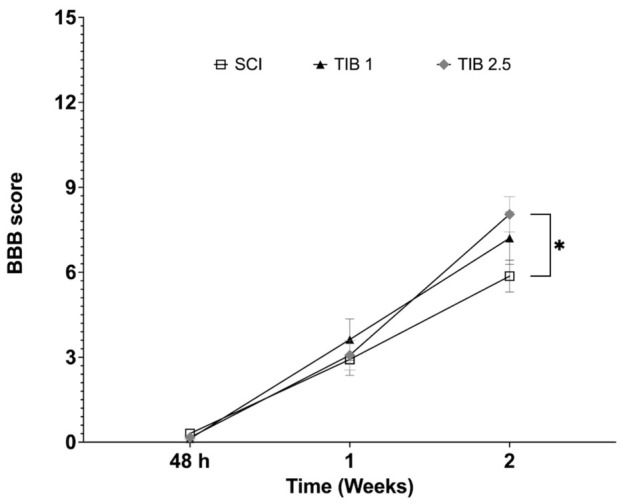
Tibolone (TIB) enhances recovery of motor function 15 days after SCI. Locomotor function was assessed using the Basso Beattie and Bresnahan (BBB) scale. Independent cohorts of animals were used (*n* = 8 per group; SCI, TIB 1, and TIB 2.5). Data were analyzed using repeated-measures one-way ANOVA with Tukey’s post hoc test. Values are presented as mean ± SEM. * *p* < 0.05 vs. SCI.

Motor function recovery was assessed using the BBB scale (Figure 5). The SCI group achieved a final average score of 5.8 ± 0.5. They exhibited consistent, albeit mild, hip, knee, and ankle movements from the second week of evaluation onward. In contrast, treatment with TIB 2.5 yielded a higher BBB score (8.0 ± 0.43), indicating greater joint mobility and partial weight support during stepping. Repeated-measures ANOVA with Tukey’s post hoc test showed a significant difference between the SCI group and TIB 2.5 (*p* = 0.0363). No significant differences were observed between SCI and TIB 1 (*p* = 0.4455) or between TIB 1 and TIB 2.5 (*p* = 0.7358).

Overall, TIB 2.5 treatment led to modest improvements in locomotor performance compared with untreated animals with SCI (Figure 5). We further evaluated whether TIB administration affected tissue preservation at the injury site. However, at 15 days post-SCI, neither TIB concentration produced a significant effect on tissue preservation compared with the untreated SCI group (Supplementary Figure S1).

## 4. Discussion

The global prevalence of SCI is estimated to affect 25–30 million people, with incidence rates ranging from 13 to 26 per 100,000 individuals and average annual costs of about $1.5 to $3 billion per country [33,34]. Despite the significant personal and economic burden linked to SCI, treatment options remain limited.

Studies have shown that estrogen and its derivatives protect against functional impairments after SCI [12,13]. However, the effects of E2 depend on the dose, and high doses can cause unwanted side effects [21,35], prompting the development of synthetic steroids such as TIB [15]. Importantly, TIB reduced oxidative stress markers 3 days after SCI when administered at 0.1 mg/kg [20,21]. Recently, oxidative stress has been identified as a key contributor to functional impairment and pathology after SCI.

In this study, we evaluated the effects of TIB at 1 mg/kg and 2.5 mg/kg on the oxidative stress marker 4-HNE at 3, 7, and 15 days after SCI. Our results showed that TIB 1 had no effect at any time point post-SCI; however, TIB 2.5 markedly decreased 4-HNE levels only at 15 days post-SCI. Previous studies reported reduced oxidative stress with a lower TIB dose (0.1 mg/kg) at 3 days post-SCI [21]. The observed differences in effective doses may reflect the biological activity of TIB, which is determined not solely by exposure to the parent compound but also by the hormonal activity of metabolites generated during intestinal and hepatic metabolism [17]. Additionally, intracellular metabolism plays a critical role in determining which steroid receptors are activated in a given tissue [36]. Ultimately, tissue responsiveness to TIB depends on the cellular context, including receptor abundance, the balance between co-activators and co-repressors [37], and the involvement of non-genomic signaling pathways such as MAPK activation [38]. However, higher doses of TIB have sometimes been shown to reduce lipid peroxidation only in specific brain regions, without producing a generalized antioxidant effect [39]. Furthermore, factors such as age and treatment duration may influence the antioxidant effects of TIB [17,39,40].

NADPH oxidase (NOX) enzymes are major sources of ROS, and several isoforms—particularly NOX2 and NOX4—are implicated in ROS production after SCI [6]. Our findings showed that NOX2 and NOX4 protein expression was detectable as early as 3 days after injury, increased significantly by day 7, and remained elevated at day 15 post-SCI. These results align with previous studies suggesting that NOX2 and NOX4 play crucial roles in ROS production in CNS injuries [9,35] and in SCI [4,6,41]. Although this study did not assess the effect of TIB at 24 h post-injury, no direct comparison with the acute peak reported in the literature can be made. However, our data are consistent with previous reports indicating an early increase in NOX2 protein expression after injury, followed by sustained elevation over time. In this regard, Byrnes et al. (2011) described a marked increase in NOX2 protein expression at 24 h post-injury, and other studies have shown that both gene and protein expression of NOX2 components (gp91 phox and p22 phox), as well as overall NOX activity, remained elevated for months following injury [4,42].

Khayrullina et al. (2023) showed that temporarily inhibiting NOX2 with intrathecal gp91ds-tat immediately after injury improved recovery in a mouse SCI model [5,6]. Additionally, knocking down NOX2 rapidly decreased ROS production and enhanced motor function. However, administration of the NOX4 inhibitor GKT137831 reduced acute oxidative stress and inflammation following moderate SCI but did not significantly improve motor function [6].

Since NOX2 inhibition improves recovery in SCI models and TIB also enhances functional recovery after SCI, we examined whether the effect of TIB is related to NOX protein expression and immunoreactivity, specifically NOX2 and NOX4. However, our Western blot results showed no statistically significant differences between the TIB-treated and untreated groups at any time point examined. These findings suggest that the observed reduction in lipoperoxidation induced by TIB 2.5 is unlikely to be mediated by changes in NOX2 or NOX4 protein levels.

It is also important to note that NOX2 produces ROS after assembling with the transmembrane protein p22 phox and the cytosolic subunits p47 phox, p67 phox, and p40 phox, as well as with one of the small Rho GTP-binding proteins, Rac1 or Rac2 [43], which was not evaluated in this study. In contrast, NOX4 is constitutively active once expressed in the membrane together with p22 phox [44,45]. Therefore, unlike NOX2, NOX4 levels are more indicative of its activation. Future studies should directly assess NOX enzymatic activity, along with the expression of all subunits and regulatory components required for each NOX isoform’s activity.

Cooney et al. (2014) demonstrated that NOX2 and NOX4 isoforms are expressed in neurons and astrocytes [4]. We therefore aimed to evaluate the specific changes in NOX immunoreactivity induced by SCI and TIB treatment in these two cell types. Our findings showed that both NOX2 and NOX4 are present in neurons and astrocytes. The highest levels of NOX2 co-localization in neurons and astrocytes occurred at 7 and 15 days post-injury in the SCI group. In previous studies, we observed that TIB at a dose of 2.5 mg/kg promoted neuroprotection after SCI [20]. The previously reported neuroprotective effects of TIB may be associated with changes in the immunoreactivity patterns of NOX2 and NOX4 in neurons and astrocytes. According to our findings, TIB at 2.5 mg/kg markedly decreased the number of NOX2-positive neurons and NOX4-positive astrocytes compared with the untreated SCI group. These findings indicate that TIB influences NOX2- and NOX4-related immunoreactivity patterns in a cell-specific manner. However, as mentioned above, inhibition of NOX4 reduced oxidative stress and inflammation following SCI but did not significantly improve motor function [6]. Although TIB treatment was associated with changes in NOX4 immunoreactivity post-SCI, the present data do not allow conclusions regarding the specific contribution of NOX4-related mechanisms to the beneficial effects observed following TIB administration.

Although NOX2 protein expression has been linked to cellular damage, NOX enzymes are also well documented to play essential roles in several physiological processes in the nervous system, including neuronal growth [46], proliferation and differentiation [47], and cerebellar development [48]. Notably, NOX2 overexpression in dorsal root ganglion neurons has been shown to promote sensory axon regeneration and improve functional recovery after spinal cord injury [49], underscoring the importance of neuron-derived NOX2 signaling in repair mechanisms.

Additionally, the effects of TIB might depend on the stage of SCI, as degenerative and regenerative cellular processes unfold at different times after injury [50,51]. This timing could influence how TIB interacts with NOX2, potentially resulting in stage-specific therapeutic effects. Many of the mechanisms by which TIB exerts its effects remain poorly understood, largely due to its distinct tissue metabolism and limited knowledge of its molecular targets, which complicate the study of its pharmacological potential. Its broad range of activity, mediated by multiple steroid receptors and involving extensive cross-talk with diverse pathways, further complicates the identification of its specific effects.

TIB has been shown to support mitochondrial function, thereby reducing oxidative damage and cell death [52]. It also lowers IL-6 levels [21,53] and suppresses pro-inflammatory markers, including IL-1β, TLR4, and Bax [52]. In addition, TIB treatment reduces reactive gliosis and decreases the overexpression of TLR4, HMGB1, NF-κB, and the NLRP3 inflammasome. These findings suggest that TIB exerts anti-inflammatory and anti-apoptotic effects, likely mediated through estrogen receptors (ERs) [52,54,55]. Additionally, TIB has beneficial effects on synaptic processes, enhancing memory across age groups [28,56]. Some of these effects appear dose-dependent; for example, TIB increased CDK5 levels at low doses but decreased them at higher doses [28], indicating concentration-dependent effects on neuronal signaling.

Functional recovery after SCI depends on multiple factors, including the cellular environment at the injury site. In this study, we investigated the effect of TIB at 15 days post-SCI. Animals treated with TIB 2.5 mg/kg exhibited wide hip, knee, and ankle movements and consistent plantar paw placement with weight support, indicating modest functional recovery compared with animals in the SCI group. This functional improvement was accompanied by reduced NOX2 immunoreactivity in neurons. Consistent with this observation, TIB 2.5 mg/kg reduced neuronal NOX2 co-localization at 3 and 7 days post-SCI. Similar findings were reported by Sabirzhanov et al. (2019), who observed comparable improvements in motor function, increased neuronal survival, and reduced ROS levels at 8 weeks post-injury in NOX2-knockout mice following SCI. Consistent with the effects of acute NOX2 inhibition, NOX2 knockout reduced ROS and improved motor function [57]. Although these observations suggest a potential relationship between neuronal NOX2-associated responses and functional outcomes after SCI, the present study does not establish a causal link between altered NOX2 immunoreactivity and motor recovery.

This study has several limitations. First, the relatively small sample size for molecular analyses may reduce statistical power to detect subtle differences in protein expression, such as NOX isoforms; however, the design avoided repeated measures in the same animals and remains consistent with exploratory SCI studies. Second, although protein levels provide indirect evidence of enzymatic modulation, they are not definitive indicators of NOX activity. Thus, mechanistic inferences regarding TIB regulation of NOX remain tentative, and future studies should directly assess NOX activity and its regulatory subunits. Third, early post-injury time points (e.g., 24 h) are critical for characterizing acute oxidative responses; nevertheless, the selected intervals allowed us to capture subacute and delayed effects of TIB. In addition, although NOX2 and NOX4 have been reported in microglia/macrophages after SCI, the present study was restricted to neurons and astrocytes. Therefore, future studies incorporating microglial and macrophage markers, along with cell-phenotype characterization, will be necessary to further clarify the cell-specific effects of TIB after SCI. Finally, the absence of an uninjured (sham) control group limits direct comparisons with baseline conditions. However, the chosen time points enabled monitoring of progressive changes during key phases of chronic SCI.

## 5. Conclusions

Our findings are consistent with previous reports that demonstrate distinct temporal and cell-specific patterns of NOX2 and NOX4 immunoreactivity in neurons and astrocytes following SCI, suggesting an association with cellular responses to injury. Importantly, TIB treatment was associated with dose-dependent changes in NOX2 and NOX4 co-localization patterns within neuronal and astrocytic populations, as evidenced by differential co-localization with NeuN and GFAP. Moreover, administration of TIB decreased oxidative stress and was accompanied by modest improvements in motor function following SCI.

Despite these observations, the present study does not allow conclusions regarding the underlying molecular mechanisms because NOX enzymatic activity, subunit assembly, and subcellular localization were not directly assessed. Future studies incorporating measurements of NOX activity, employing selective NOX inhibitors, and genetic loss-of-function approaches will be essential to clarify this relationship and determine whether NOX-related pathways contribute to the cellular and functional effects associated with TIB treatment after SCI.

## Data Availability

The original contributions presented in this study are included in the article. Further inquiries can be directed to the corresponding author(s).

## Supplementary Materials

The following supporting information can be downloaded at: https://www.mdpi.com/article/10.3390/brainsci16070711/s1.

## Abbreviations

The following abbreviations are used in this manuscript:

BBB	Basso, Beattie, Bresnahan
GFAP	Glial fibrillary acidic protein
4-HNE	4-hydroxynonenal
NADPH	Nicotinamide adenine dinucleotide phosphate
NOX2	NADPH oxidase 2
NOX4	NADPH oxidase 4
OS	Oxidative stress
ROS	reactive oxygen species
SCI	Spinal cord injury
TIB	Tibolone
