# Supplementary material for: Tibolone Administration Is Associated with Enhanced Motor Recovery and Decreased Cell-Specific NOX2 and NOX4 Immunoreactivity in a Rat Model of Traumatic Spinal Cord Injury
Source: Brain Sci. 2026 Jul 1;16(7):711. doi: 10.3390/brainsci16070711 (PMC13406349; doi:10.3390/brainsci16070711)
Supplement: Supplementary file 1 [file brainsci-16-00711-s001.zip › brainsci-4275961-supplementary.pdf]

Supplementary Figure S1

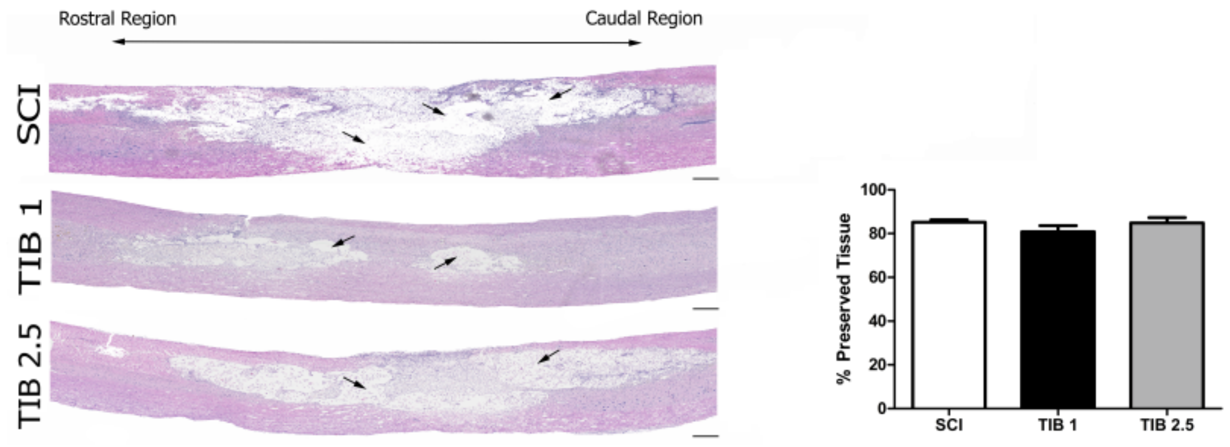

Representative images of longitudinal sections of the spinal cord stained with hematoxylin-eosin 15 days post-surgery: SCI, Tib 1 (tibolone 1 mg/kg), and Tib 2.5 (tibolone 2.5 mg/kg) groups. Images correspond to the rostral area, the epicenter of the injury, and the caudal area (panoramic 2× magnification). (B). Quantification of % preserved tissue of a 2 cm segment of the spinal cord, including the rostral region, the epicenter of the lesion, and the caudal region, after 15 days of surgery in the SCI or tibolone-treated (Tib 1 and Tib 2.5) animals. Data are presented as the mean ± SE % of preserved tissue (n = 4). Data were analyzed using one-way ANOVA, followed by the Tukey post hoc test ($p < 0.05$).
